# Visualization of sympathetic neural innervation in human white adipose tissue

**DOI:** 10.1098/rsob.210345

**Published:** 2022-03-16

**Authors:** Aliki Perdikari, Tessa Cacciottolo, Elana Henning, Edson Mendes de Oliveira, Julia M. Keogh, I. Sadaf Farooqi

**Affiliations:** Wellcome-MRC Institute of Metabolic Science, University of Cambridge, Addenbrooke's Hospital, Cambridge, UK

**Keywords:** obesity, human adipose tissue, sympathetic innervation, whole tissue immunolabelling, three-dimensional microscopy

## Abstract

Obesity, defined as an excess of adipose tissue that adversely affects health, is a major cause of morbidity and mortality. However, to date, understanding the structure and function of human adipose tissue has been limited by the inability to visualize cellular components due to the innate structure of adipocytes, which are characterized by large lipid droplets. Combining the iDISCO and the CUBIC protocols for whole tissue staining and optical clearing, we developed a protocol to enable immunostaining and clearing of human subcutaneous white adipose tissue (WAT) obtained from individuals with severe obesity. We were able to perform immunolabelling of sympathetic nerve terminals in whole WAT and subsequent optical clearing by eliminating lipids to render the opaque tissue completely transparent. We then used light sheet confocal microscopy to visualize sympathetic innervation of human WAT from obese individuals in a three-dimensional manner. We demonstrate the visualization of sympathetic nerve terminals in human WAT. This protocol can be modified to visualize other structures such as blood vessels involved in the development, maintenance and function of human adipose tissue in health and disease.

## Graphical abstract

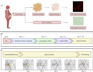

## 1. Introduction

Obesity-related complications such as type 2 diabetes and fatty liver disease represent a significant disease burden. As people gain weight, white adipose tissue (WAT) mass expands to store energy-rich triglycerides. Studies in rodents and in humans with inherited disorders of adipose tissue development have shown that there is considerable variability in how much adipose tissue expansion can take place [1]. Once the adipose tissue expansion limit is reached, adipose tissue ceases to store triglycerides efficiently and lipids begin to accumulate ectopically in other tissues and organs such as the liver and skeletal muscle, causing lipotoxicity, insulin resistance, cellular apoptosis and inflammation, mechanisms that underpin the development of obesity-associated metabolic complications [2].

To date, much of our understanding of adipocyte biology has emerged from histological studies that have revealed changes in adipocyte cell size, extracellular matrix (ECM) flexibility and fibrosis, and macrophage infiltration in obesity [3], while the development of whole-mount immunolabelling has provided insights into adipocyte precursor cell development [4]. A major function of adipose tissue is the release of stored triglycerides in WAT by lipolysis in times of nutrient insufficiency, a process that is regulated by activation of the sympathetic nervous system. However, the neural innervation of WAT has been difficult to visualize due to the presence of large cells filled with lipids [5]. In classical studies in rodents, Bartness and colleagues used retrograde tracers to perform neuronal tracing between adipose tissue and the brain in order to determine the central circuits that innervate various adipose tissue depots [6]. Recent studies in mice have shown for the first time that white adipocytes are directly innervated by sympathetic neurons and that sympathetic innervation of WAT is necessary and sufficient to drive leptin-mediated lipolysis [7,8]. Further studies using whole tissue immunolabelling, optical clearing and three-dimensional volume microscopy have revealed important information regarding the innervation of mouse adipose tissue and pathways responsible for the regulation of sympathetic innervation [9]. Here we set out to optimize optical clearing techniques and whole-mount immunolabelling protocols used in mice to visualize sympathetic neural circuits innervating WAT in humans. By contrast to optical clearing and whole tissue immunolabelling protocols previously described in mice [7,10], the protocol reported here uses inexpensive, simple-to-make and non-hazardous solutions which are compatible with different light sheet confocal microscopes, enabling the analysis of human adipose tissue morphology and neural innervation.

## 2. Results

### 2.1. Subcutaneous adipose tissue from obese individuals retains most of its original biopsy size during whole mount immunolabelling and optical clearing

**Figure 1.**
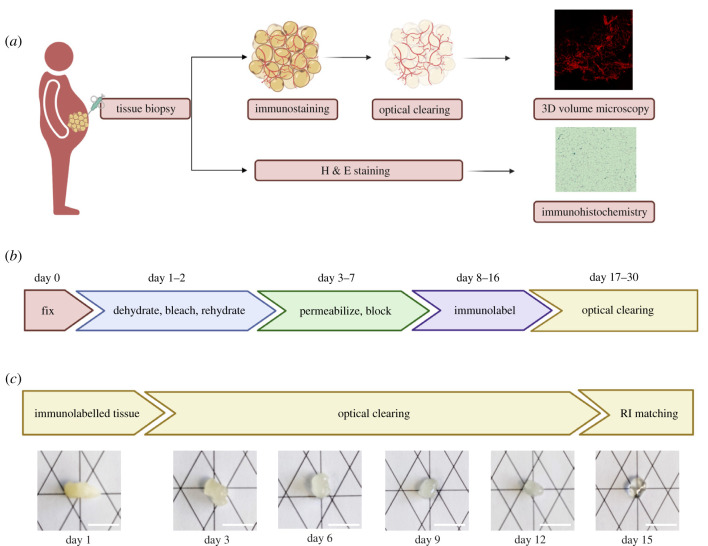
Processing of human adipose tissue (*a*) Schematic representation of processing of human adipose tissue including immunolabelling, optical clearing and three-dimensional volume imaging as well as haematoxylin and eosin (H & E) staining. (*b*) Schematic representation of the immunolabelling protocol for human adipose tissue. (*c*) Representative images for the optical clearing process (ScaleCUBIC 1 and ScaleCUBIC 2) for human adipose tissue at different time-points, scale bar: 1 cm.

We performed open biopsies of abdominal subcutaneous adipose tissue from individuals with severe obesity. Two grams of adipose tissue were immediately placed on dry ice and cut into two pieces. One piece was immersed in 4% formaldehyde (FA) and 10% sucrose in phosphate buffer saline (PBS) and kept at 4°C for 24 h for whole-mount immunolabelling, optical clearing and three-dimensional volume imaging. The second piece was immersed in 10% neutral buffered formalin solution (NBF) and kept at room temperature for histology and haematoxylin and eosin staining (H & E) (figure 1*a*). We then optimized the iDISCO technique for whole-mount immunolabelling, which is based on dehydration and rehydration of the tissue in methanol to increase adipose tissue permeability [7,11]. After multiple rounds of organic solvent-based dehydration/rehydration to remove lipids and enhance tissue permeabilization, bleaching with hydrogen peroxide to reduce tissue auto-fluorescence was performed, followed by immunolabelling with primary antibody against tyrosine hydroxylase (TH) and secondary antibody, and optical clearing with hyperhydrating solutions (figure 1*b*). Although during dehydration the human adipose tissue biopsy reduced in size, it regained most of its original size during the rehydration steps. A small change in size was observed by the end of the dehydration/rehydration process, as measured by tissue area; further analysis is needed to assess whether there was a significant change in tissue volume (electronic supplementary material, figure S1). Moreover, while the tissue became more rigid during the whole process it regained its original texture upon incubation for 1 h in phosphate-buffered saline (PBS) and during subsequent washing, permeabilization and blocking steps. For immunolabelling, we used an antibody against TH, which is the rate-limiting enzyme of catecholamine biosynthesis and a marker for sympathetic nervous system neurons. Since lipids are a major source of auto-fluorescence, we used Alexa-568 fluorophore in the red spectrum as a secondary antibody, avoiding blue-green fluorophores, which generate more scatter when passing through tissue.

### 2.2. Whole adipose tissue optical clearing performed using the advanced CUBIC method

We performed adipose tissue clearing using the advanced CUBIC method, which is based on simple immersion in two different reagents to minimize light scattering [12]. Tissue was immersed in ScaleCUBIC-1 reagent, which can remove lipids, the main light scattering material inside the tissue and ScaleCUBIC-2 a reagent to further minimize light scattering and match the refractive indices (RIs) between the sample and the reagent, minimizing reflection or refraction and leading to a higher resolution image (figure 1*c*). Tissue texture was altered after optical clearing, leading to a more gel-like consistency due to the clearance of lipids, changing the shape of the tissue as well as its size (electronic supplementary material, figure S1), as reported for other tissues cleared with the CUBIC protocol [13].

### 2.3. Optical clearing with the CUBIC protocol retains adipose tissue morphology and increases whole adipose tissue light transmittance

**Figure 2.**
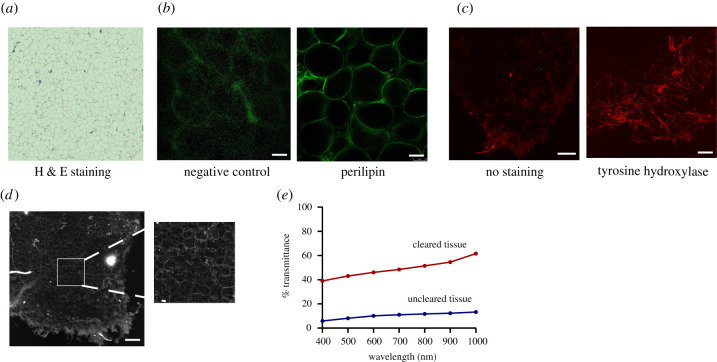
Morphology and light transmittance of human adipose tissue biopsies. (*a*) Representative haematoxylin and eosin (H & E) staining of human adipose tissue depicting lipid droplets within adipocytes. (*b*) Representative confocal image of human adipose tissue before clearing; adipocytes stained with perilipin (green) compared to secondary antibody only (negative control); scale bar, 100 μm. (*c*) Maximum intensity projection images of cleared human adipose tissue without (left) or with (right) tyrosine hydroxylase (TH) staining obtained using a light sheet microscope. Red: tyrosine hydroxylase, scale bar: 200 μm. (d) Maximum intensity projection image of autofluorescence of cleared human adipose tissue, scale bar: 200 μm. (*e*) Quantification of light transmittance of uncleared and optically cleared adipose tissue. Data presented as percentage transmittance, mean of three individual measurements of the same tissue, normalized to blank.

We used H & E staining of tissue sections to confirm the presence of intact adipocytes comprised large lipid droplets (figure 2*a*). Moreover, we showed that the adipose tissue remained intact after the biopsy as indicated by the presence of adipocytes (perilipin staining) and imaging with a conventional confocal microscope (figure 2*b*). After immunolabelling and optical clearing of human adipose tissue, we tested the specificity of the TH antibody to label sympathetic neural arborizations. We observed immunolabelling of neural connections in the optically cleared adipose tissue incubated with the TH antibody in comparison with tissue incubated only with the secondary antibody (figure 2*c*). Furthermore, using the auto-fluorescent properties of human adipose tissue, we could show that the overall tissue architecture remained intact even after the lengthy protocol of permeabilization, immunolabelling and clearing (figure 2*d*). Lastly, we examined light transmittance in the optically cleared whole adipose tissue after using the CUBIC protocol to assess the degree of tissue clearance (figure 2*e*). We could show that there is an increase of about 40% in light transmittance in the cleared sample in comparison to the light transmittance observed prior to optical clearing.

### 2.4. Quantification of sympathetic neural innervation using whole adipose tissue three-dimensional images

**Figure 3.**
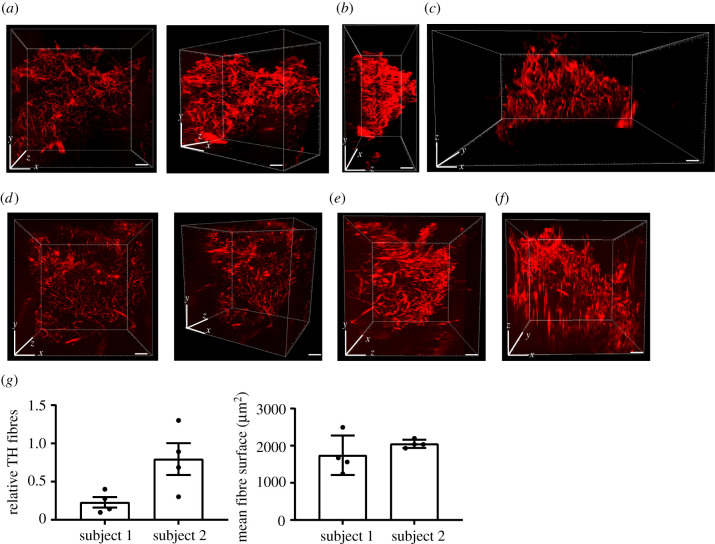
Imaging of sympathetic neural arborizations in human adipose tissue. (*a*–*f*) Representative images of the neural network in human adipose tissue obtained with light sheet microscopy from two subjects. (*a*,*d*) *xy* plane; (*b*,*e*) *yz* plane; (*c*,*f*) *xz* plane; scale bar: 1 mm. (*g*) Quantification of TH fibres presented as relative TH fibres and fibre surface, *n* = 4 tissue areas, mean ± standard deviation (error bars).

We analysed cleared immunolabelled tissue from two severely obese people stained for TH using a light sheet confocal microscope that revealed in three dimension the presence of sympathetic neural networks in human adipose tissue (figure 3*a–f*; electronic supplementary material, movies S1 and S2). We used the three-dimensional images obtained to calculate the density of neural arborizations (relative number of TH+ fibres) and the mean fibre surface (figure 3*g*), which revealed variation in fibre size between subjects and also between different areas within the same piece of tissue (electronic supplementary material, figure S2).

## 3. Discussion

Until recently, the ability to study adipose tissue innervation has been limited by the technical properties of conventional microscopy. For several decades there has been doubt as to whether direct innervation of adipose tissue exists. The combination of tissue clearing techniques with immunolabelling and advanced three-dimensional volume microscopy has made it possible to image whole organs and study sympathetic innervation in murine adipose tissue at single fibre resolution [7–10]. In this study, we obtained human adipose tissue biopsies from severely obese people and developed a protocol for immunolabelling, optical clearing and three-dimensional visualization of neural networks within human WAT using a light sheet confocal microscope. We quantified the density of TH positive fibres in human adipose tissue samples by analysing the three-dimensional images obtained. Although different protocols exist for whole adipose tissue immunostaining and three-dimensional imaging of mouse adipose tissue [7,10], this is the first report of successful three-dimensional imaging of sympathetic neuronal connections in human adipose tissue. Further studies of small sections of adipose tissue at higher magnification will be needed to quantify innervation by fine nerve fibres.

For optical clearing of human WAT, we optimized the advanced CUBIC protocol, which is based on delipidation and hyperhydration of the tissue followed by RI matching. The advantage of using the CUBIC method, despite the lengthy protocol required and decreased clearance performance for large amounts of tissue in comparison to other protocols [12], is that the solutions used are based on urea and Triton X-100, and are non-hazardous, compared to organic solvents used in the benzyl alcohol/benzyl benzoate (BABB), AdipoClear and iDISCO protocols used for murine adipose tissue clearing [10,11,14]. A major disadvantage of this protocol is that clearing of the tissue takes several days, which may decrease staining intensity; notably, the tissue becomes highly fragile and difficult to handle in the clearing solution. The use of smaller samples would minimize the time needed for optical clearing.

This technique can be applied to study human adipose tissue biology and address important questions such as the differences between lean and obese people, the role of leptin in adipose tissue innervation, and the impact of weight loss on adipose tissue structure and function. There are a number of technical considerations when adapting this protocol for studies in larger clinical cohorts. Prior to immunolabelling, an antibody needs to be tested for compatibility with the methanol present in the dehydration/rehydration steps of the tissue, while antibody concentration may need further adjustment. Additionally, the size of the WAT sample will determine the time needed for effective permeabilization and blocking, as well as the length of incubation with the primary and secondary antibody. For the tissue mass used here, we required 5 days of permeabilization and blocking and 8 days of incubation in the antibody solutions. We obtained human adipose tissue biopsies surgically, which retained the morphology of the adipose tissue. We did not try the same protocol with adipose tissue obtained by other means, such as using fine needle biopsies, where the needle bore and application of suction may damage tissue structure. Moreover, we obtained tissue from severely obese people, where the amount of lipid and presence of tissue inflammation/fibrosis (compared to lean people) may have impacted on immunolabelling and tissue clearing. We observed a reduced antibody signal in the centre of the adipose tissue biopsy (electronic supplementary material, movie S1) that probably results from decreased antibody penetration caused by incomplete clearing of lipids. Studies using smaller samples and increased antibody concentration may improve clearing and staining.

In summary, we have developed a protocol for the optical clearing and immunolabelling of human WAT that permits the direct visualization and quantification of sympathetic neural innervation for the first time. Given the importance of sympathetic neural connections in mediating lipolysis and potentially other functions, the method proposed here represents a powerful tool to further understand human adipose tissue structure and function, and its disturbance in obesity and metabolic disease.

## 4. Material and methods

### 4.1. Subjects

Both male participants had a history of severe childhood obesity. At the time of the study, subject 1 was 22 years old with a BMI of 40.6 kg m^−2^, subject 2 was 24 years old with a BMI of 42.7 kg m^−2^. They had refrained from smoking, alcohol, caffeine and strenuous exercise for 24 h prior to the biopsy. Biopsies were carried out 3 h after the consumption of a standardized lunch (35% of total energy requirements; 50% carbohydrate, 30% fat, 20% protein). Studies were performed at the Wellcome MRC Institute of Metabolic Science Translational Research Facility.

### 4.2. Adipose tissue biopsy

Subcutaneous adipose tissue biopsies were obtained from the left iliac fossa, using a non-diathermy surgical biopsy method, under local anaesthesia. Participants were positioned in the supine position on a surgical couch. The skin was cleaned with 2% chlorhexidine in 70% isopropyl alcohol (Chloraprep) and covered with surgical drapes to create a sterile field. The skin was infiltrated with a maximum of 10 ml local anaesthetic (1% lidocaine with 1 : 200 000 adrenaline). A 2–3 cm skin incision was made, skin flaps were raised on either side of the incision and the superficial fascia incised. Adipose tissue was grasped with atraumatic forceps; approximately 2 g of tissue was excised and washed in PBS. Haemostasis was achieved using manual compression. A subcuticular suture (3/0 Vicryl Rapid 19 mm) was passed and reinforced with histoacryl tissue adhesive.

### 4.3. Adipose tissue preparation

For whole-mount immunolabelling adipose tissue biopsies were fixed in 20 ml PBS with 4% formaldehyde (Fisher Chemical) and 10% sucrose (Sigma) in a 50 ml falcon tube overnight at 4°C with gentle shaking. Tissue was then washed 3 times with 5 ml PBS for 1 hour each time at room temperature and then either processed immediately for whole-mount immunolabelling, or kept in 70% ethanol at 4°C for maximum one month. For histology and haematoxylin and eosin staining (H & E) adipose tissue biopsies were fixed in 10% NBF in 25 ml histology pots at room temperature until further processing.

### 4.4. Whole-mount tissue immunolabelling

The whole-mount immunolabelling protocol was adapted from Jiang *et al.* [7]. Fixed adipose tissue was cut into 1–2 cm^3^ pieces. All steps were performed in 2 ml Eppendorf tubes unless stated otherwise. Adipose tissue dehydration was performed at room temperature, with gentle shaking and serial incubation in 1.5 ml 20% methanol (Sigma) in double-distilled water (ddH_2_O) for 30 min, 40% methanol in ddH_2_O for 30 min, 60% methanol in ddH_2_O for 30 min, 80% methanol in ddH_2_O for 30 min and twice in 100% methanol in ddH_2_O for 30 min. Dehydrated tissue was then bleached with gentle shaking at 4°C for 48 h in 1.5 ml of 5% H_2_O_2_ (Sigma) in methanol (1 volume of 30% H_2_O_2_ in 5 volumes of 100% methanol) supplemented with 10 mM EDTA and adjusted for pH 8 with Na. Tissue rehydration was performed at room temperature and with gentle shaking by serial incubation in 1.5 ml 80% methanol in ddH_2_O for 30 min, 60% methanol in ddH_2_O for 30 min, 40% methanol in ddH_2_O for 30 min, 20% methanol in ddH_2_O for 30 min and twice in 1.5 ml PBS supplemented with 0.2% Triton X 100 (Fisher Chemical) for 1 h. Tissue was permeabilized in 1.5 ml of PBS with 0.2% Triton X 100, 20% dimethyl sulfoxide (DMSO) (Sigma) and 0.3 M glycine (Sigma) at 37°C for 48 h with gentle rotation and then blocked in 1.5 ml of PBS with 0.2% Triton X 100, 10% DMSO and 5% donkey serum (D9663 Sigma) at 37°C for 72 h with gentle rotation. Tissue was then immunolabelled with 1 : 100 anti-rabbit TH antibody (Millipore, AB152) or 1 : 500 anti-goat perilipin antibody (Abcam, ab61682) in 1.5 ml of PBS with 0.2% Tween20 (Sigma), 5% DMSO, 5% donkey serum and 10 µg ml^−1^ heparin (Sigma) at 4°C for 96 h with gentle rotation, washed five times in 1.5 ml of PBS with 0.2% Tween20, and 10 µg ml^−1^ heparin for 1 h each time with gentle rotation and labelled with the secondary antibody donkey anti-rabbit Alexa 568 (Invitrogen, A-31573) or donkey anti-goat Alexa 488 (Invitrogen, A-11055) in 1.5 ml of PBS with 0.2% Tween20, 5% DMSO, 5% donkey serum and 10 µg ml^−1^ heparin at 4°C for 96 h with gentle rotation. Tissue was washed 5 times in PBS with 0.2% Tween20 and 10 µg ml^−1^ heparin at room temperature for 2 h each time with gentle rotation and then kept in 1.5 ml PBS at 4°C until optical clearing.

### 4.5. Tissue optical clearing and refractive index match

The optical clearing protocol was adapted from Susaki *et al.* [12]. In detail, ScaleCUBIC1 (reagent 1) was prepared by mixing 25 wt% (weight percent = weight of solute/weight of solvent × 100) final concentration Urea (Sigma), 25 wt% final concentration Quadrol 80% (Aldrich Chemical) and 15 wt% final concentration Triton X 100 in ddH_2_O and kept at room temperature for maximum one month. ScaleCUBIC2 (reagent 2) was prepared by mixing 25 wt% final concentration Urea, 50 wt% final concentration sucrose (Sigma) and 10 wt% final concentration triethanolamine (Aldrich Chemistry) in ddH_2_O and kept at room temperature for maximum one month. All steps for optical clearing were performed in 2 ml Eppendorf tubes. Immunolabelled adipose tissue was incubated in 1.5 ml 1 : 1 Reagent 1/ddH_2_O solution at 37°C for 3–6 h with gentle rotation and then transferred in 1.5 ml Reagent 1 solution and incubated at 37°C with gentle rotation between 8 and 15 days. Reagent 1 solution was refreshed every second day until tissue looked clear. After that, adipose tissue was incubated in 1.5 ml 1 : 1 Reagent 2/PBS solution at 37°C for 3–6 h with gentle rotation and then transferred in 1.5 ml Reagent 2 solution at 37°C for 72 h with gentle rotation until imaged in the microscope.

### 4.6. Measurement of tissue light absorbance, calculation of transmittance and measurement of tissue biopsy area

Uncleared and cleared adipose tissue was immersed in 100 µl of CUBIC2 reagent in a 96-well clear-bottomed microplate (Greiner). Tissue absorbance measurements were taken every 2 nm between 400 nm and 1000 nm with the Spark M10 Microplate Reader (Tecan). Blank measurements were taken from an equal volume of CUBIC2 reagent in the same 96 well plates. To calculate tissue transmittance (*T*) from absorbance (*A*) we used the equation *T* = 10^(−*A*) and calculated the mean of three individual measurements of the same tissue. Results are presented as percentage of normalized transmittance to blank, which is set at 100%.

Measurement of tissue biopsy area before, during and after dehydration and rehydration steps and optical clearing was made with ImageJ. The length of the scale bar in the images (1 cm) was used to set the reference scale for measurements. The tissue perimeter was drawn using the area selection tool and the area was calculated in cm^2^ using the measure function.

### 4.7. Three-dimensional volume fluorescent imaging, confocal imaging, image processing and density quantification

Optically cleared adipose tissue was imaged on a Zeiss Z1 light sheet microscope in clearing configuration using a single or a dual-side illumination, with the EC Plan-Neofluar 5×/0.16 Air immersion objective, a 561 nm laser illumination and the BP 575-615 nm emission filter. Tissue was attached with glue in the edge of a syringe in order to be immobilized and immersed in the imaging chamber filled with ScaleCUBIC 2 (Reagent 2) solution carefully to avoid bubble formation. Acquisition and initial image processing were performed using ZEN black software (Zeiss, 2014 service pack 1 v. 9.2.6.54), stitching was performed in Arivis software (Arivis) and further image processing in Fiji and ZEN lite (Zeiss). Imaging of perilipin in uncleared adipose tissue was performed using a Leica SP8 confocal microscope (Leica Microsystems).

Quantification of TH fibre density and fibre surface (μm^2^) was performed using the Arivis Vision4D software (Arivis). Fibre volume was calculated (μm^3^) in 4 Z-stack areas per subject using the fluorescent signal and the ‘blob finder’ function. The diameter of fibres was calculated (blob finder) and the threshold was adjusted for object segmentation and splitting sensitivity to identify fibres. As a last step, the volume of all objects identified was calculated. Fibres with volume less than 600 µm^3^ were not included in the analysis. Fibre volume (μm^3^) was divided by total volume (μm^3^) to calculate the relative proportion of TH fibres. Tissue surface area (μm^2^) was calculated with ImageJ (Fiji). Graphs were prepared with Prism 7 (Graph Pad Software).

### 4.8. Graphic representations

Schematics used in the figures and supplementary figures were created with Biorender (BioRender.com).

## Data Availability

All data supporting our research are included in the submission.
